# Impact of childhood trauma on sensory gating in patients with first-episode schizophrenia

**DOI:** 10.1186/s12888-018-1807-7

**Published:** 2018-08-16

**Authors:** Xian-Bin Li, Qi-Jing Bo, Qing Tian, Ning-Bo Yang, Zhen Mao, Wei Zheng, Yu-Jie Wen, Chuan-Yue Wang

**Affiliations:** 1 The National Clinical Research Center for Mental Disorders & Beijing Key Laboratory of Mental Disorders & Beijing Institute for Brain Disorders Center of Schizophrenia, Beijing Anding Hospital, Capital Medical University, No.5 Ankang Lane, Dewai Avenue, Xicheng District, Beijing, 100088 China; 2 The Affiliated Brain Hospital of Guangzhou Medical University (Guangzhou Huiai Hospital), Guangzhou, China

**Keywords:** Childhood trauma, Sensory gating, Schizophrenia

## Abstract

**Background:**

Childhood trauma (CT) has been found to contribute to the onset of schizophrenia and auditory sensory gating deficit is a leading endophenotype for schizophrenia. However, the association between the CT and sensory gating in first-episode schizophrenia remains elusive.

**Methods:**

Fifty-six patients and 49 age and sex-matched healthy controls were assessed using the Childhood Trauma Questionnaire-Short Form (CTQ-SF) for CT and Positive and Negative Syndrome Scale (PANSS) for symptoms severity. Sensory gating was tested using the modified paradigm, perceived spatial separation-induced prepulse inhibition (PSS-PPI), and the perceived spatial co-location PPI (PSC-PPI or classical PPI).

**Results:**

Comparing with healthy controls, the patients had significantly higher score on sexual abuse (*t* = 2.729, *p* < 0.05), lower PSS- PPI, % (ISI = 120 ms and ISI = 60 ms) (*t* = − 3.089, − 4.196, *p* < 0.05). Univariate analysis revealed the absence of a significant correlation among CT, PPI paradigms and symptoms. However, multiple linear regression analyses demonstrated the CTQ-SF total was negatively associated with PSS PPI (ISI = 120 ms) (*p* = 0.018).

**Conclusion:**

The current study illustrates that the impact of CT on sensory gating in patients with first-episode schizophrenia, and thus we conclude that CT may be a risk factor to the occurrence of schizophrenia through its impact on sensory gating.

## Background

Schizophrenia can be understood as a complex illness of adaptation to social context. Although heritability is often emphasized, the fact that occurrence is associated with environmental factors such as childhood trauma (CT) suggests that exposure to life stress may play an important role in developing “social” brain during sensitive periods [1]. Childhood adversities, particularly exposure to multiple adversities involving hostility, threat, and CT (emotional, physical, and sexual abuse; emotional and physical neglect) have been found to contribute to the onset of psychiatric disorder including schizophrenia [2–4].

Auditory sensory gating deficit is a leading endophenotype for schizophrenia [5]. The prepulse inhibition (PPI) is the weakening of acoustic startle reflex (ASR) when a weaker sensory stimulus precedes the startling stimulus [6], which is considered as a good indicator of sensory gating [7]. PPI impairment has been found to always be associated with core features of schizophrenia, such as aggressive behavior [8] and positive symptoms [9, 10], and relatively stable across treatment conditions [11]. It is also considered as an early sign or residual symptom of schizophrenia [12, 13].

The social isolation rat model shares some similarities with the humans experiencing CT and some neurobiological changes in socially isolated rats can mimic characteristics of schizophrenia, therefore it can serve as a model of schizophrenia [14]. Studies show that socially isolated rats exhibit increased level of ASR and produce specific deficits in PPI [6, 15]. Furthermore, exposure to CT during puberty was associated with increased risk for neuropsychiatric disorders through PPI deficit in rats [16].

In the previously study, we found sexual abuse can be a predisposing factor that affects sensorimotor gating in the patients with chronic schizophrenia [17]. To date, the association between the ASR, PPI and CT in first-episode schizophrenia has not been explored. Therefore, we aimed to examine the relationship between CT, ASR and PPI in Chinese patients diagnosed with first-episode schizophrenia in the current study.

## Methods

### Subjects

All participants were inpatients or outpatients of Beijing Anding Hospital, Capital Medical University in Beijing, China. The inclusion criteria were: (1) Met the diagnostic criteria of first-episode (less than 60 months) schizophrenia based on the Structured Clinical Interview for DSM-IV (SCID) [18]; (2) Had been clinically stable for > 12 weeks; (3) had a IQ above 80 on the Wechsler Adult Intelligence Scale (CWAIS) [19] and were able to read, comprehend and sign the consent. Patients were excluded if they were clinically unstable. In addition, 49 individuals without severe mental disorders or current substance use (included nicotine abuse) were recruited from the same geographical areas to serve as healthy controls for the PPI test battery and CTQ assessment (Controls’ age mean ± SD: 26.2 ± 3.9; years of education mean ± SD:14.0 ± 2.3).

The protocol of this study was revised and approved by the ethics committee of Beijing Anding Hospital, Beijing, China. We followed the Declaration of Helsinki while we conducting the research. Each patient signed an informed written consent, an overseeing mental health expert have ruled that all adult patients and participants have been capable of ethically and medically consenting for their participation in the research.

In the case of members of this study may not be capable of providing ethical consent for their participation, we would provide a legal guardian or representative to provide consent to participate in their stead. A total of 62 eligible patients between the age of 16 and 65 years old were initially enrolled, however, 10 patients (10.7%) dropped out from the study.

### Assessment of childhood trauma questionnaire – Short form (CTQ-SF)

We used the CTQ-SF to screen for CT in our patient and the healthy controls. The CTQ-SF is a 28-item retrospective self-report survey of CT experiences, which has five categories for children 12 years and older [20]. The five subscales included were emotional abuse (EA), physical abuse (PA), sexual abuse (SA), emotional neglect (EN) and physical neglect (PN). Each subscale contains five items [21]. The Chinese version of CTQ-SF has been shown to have good validity and reliability [22].

### Assessment of psychiatric symptomatology and demographic features

We used the Positive and Negative Symptoms Scale (PANSS) to evaluate the symptom of the patients [23]. Furthermore, we used locally-developed data collection tables to collect demographic characteristics (age at onset, age of first treatment seeking, the duration of untreated psychosis).

### Assessment of startle reflex

#### Apparatus

The experiments was conducted in a sound shielded room, and the temperature and humidity was comfortable. Two Ag/AgCl electrodes (diameter, 0.4 cm; resistance <5kΩ) were positioned on below and lateral to the right eye, over the orbicularis oculi muscle. Acoustic startle reflection was measured as the eyeblink component from EMG activity (filtering, 100–1000 Hz; amplifying, 10,000). Acoustic signals were delivered binaurally through Sennheiser HD 600 headphone. Acoustic sound intensity were calibrated by AUDit and System 824 audiometer calibration (Larson Davis, USA).

#### Testing procedure

**Fig. 1:**
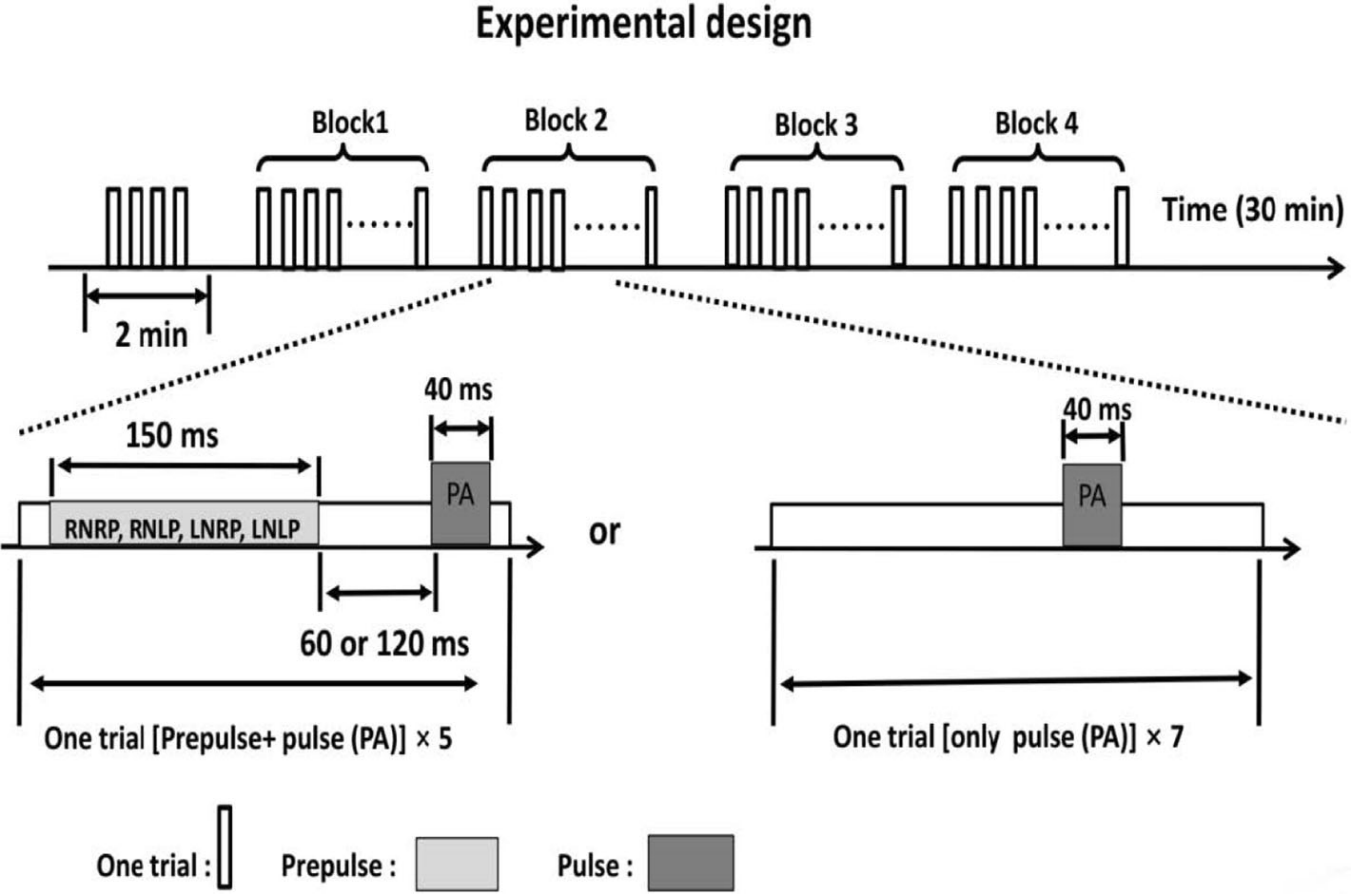
Schematic illustration of the prepulse inhibition paradigm. In a block design (7 min), a background wideband noise was continuously delivered as the masker (left leading or right leading), 7 trials contained the startling (pulse) sound alone, and 20 trials contained the prepulse (left leading or right leading) 120 ms or 60 ms preceding the startling (pulse) noise. Trials in each block were presented randomly with the inter-trial interval about 20 s. Note: RNRP (RNLP): right leading masking with prepulse co-location (separation); LNLP (LNRP): left leading masking with prepulse co-location (separation). We have published this paradigm in our privous paper ([24])

A new designed paradigm was used in PPI testing. The prepulse sound (acoustic intensity, 65 dB SPL; duration, 150 ms; broadband white noise) was performed from headphones with interaural leading time differences at each ear onset delay 3 ms (left ear leading or right ear leading). Moreover, the background noise (acoustic intensity 60 dB SPL from 0 to 10 kHz) was presented in this testing as a masker (interaural leading time differences, 3 ms), and the background noise was contributed to a fused noise-masker signal between prepulse sound (target sound) and the background noise (masker). As a result of precedence effect, two types of perceived spatial relationships were generated: perceptual spatial separation PPI (PSS PPI) and perceptual spatial co-location PPI (PSC PPI). A trial performed a prepulse stimuli, followed by a startling pulse stimuli of 104 dB SPL broadband white nosie for 40 ms. Two lead inter-stimulus interval (ISI) (prepulse onset to pulse onset) were used (60 ms, 120 ms). Then a new trial started in a random time (15 to 25 s). Thus, four trial combinations (PSC PPI or PSS PPI * ISI 120 ms or 60 m’s) were performed in the experiment. We described the detailed measurement procedures in our other published articles (Fig. 1) [24].

### Statistical analysis

We used student’s t-test to compare the startle activity, PPI and CT in patients and healthy controls. Spearman’s correlation was used to evaluate the correlation among symptoms, PPI and CT. In addition, a multiple linear regression analysis was conducted to investigate the association between childhood trauma and PPI. We used the Statistical Package of Social Sciences (SPSS, version 16.0) to conduct the statistical analyses.

## Results

### Demographics features and CT

This study included 56 patients with first-episode schizophrenia (22 males and 34 females) with an average age 25.9 ± 6.8 years (). The duration of untreated psychosis was 20.33 ± 18.14 months (1 to 60). Forty-nine healthy controls were recruited from the same geographical areas. The patients had higher score of SA than health controls (actual scores 6.47 vs 5.50, *t* = 2.729, *p* < 0.05). The patients had lower PSS PPI (both ISI = 120 ms, ISI = 60 ms) than healthy controls (*t* = − 3.089, − 4.196, *p* < 0.05, respectively). Demographic data and clinical features of the enrolled patients are summarized in Table 1.

**Table 1:** Demographic and clinical characteristics of schizophrenia patients and controls

	Schizophrenia patients (*N* = 56)	Controls (*N* = 49)	X^2^/t	*P*
Gender, M/F	22/34	25/24	1.442	0.245
Age, years	25.90 ± 6.80	26.21 ± 3.90	− 0.274	0.785
Education, years	13.18 ± 3.24	14.12 ± 3.27	−1.372	0.173
DUP, months	29.92 ± 29.63			
Drug-naïve/ on medication	24/32			
	Risperidone 10			
	Paliperidone 6			
	Olanzapine 7			
	Aripiprazole 6			
	Quetiapine 3			
On medication>2w/<2w	11/21			
PANSS				
Total	82.28 + 22.32			
Positive	22.57 ± 7.17			
Negative	19.25 ± 9.33			
General Psychopathology	43.02 ± 5.57			
Childhood trauma				
Total	42.89 ± 11.60	38.60 ± 8.86	1.919	0.058
Emotional abuse	7.97 ± 3.22	7.00 ± 2.48	1.589	0.116
Physical abuse	6.11 ± 2.15	6.26 ± 2.08	− 0.314	0.754
Sexual abuse	6.47 ± 2.13	5.50 ± 1.06	2.729	0.008
Emotional neglect	12.83 ± 5.04	10.97 ± 3.94	1.910	0.060
Physical neglect	9.87 ± 3.16	8.86 ± 2.31	1.679	0.097
PPI index				
Startle	77.41 ± 36.82	74.73 ± 30.33	0.322	0.748
PPI (ISI = 120 ms), %				
PSC PPI	20.20 ± 25.86	27.77 ± 20.31	−1.321	0.191
PSS PPI	21.06 ± 23.13	37.82 ± 20.58	−3.089	0.003
PPI (ISI = 60 ms), %				
PSC PPI	19.98 ± 19.60	28.45 ± 18.57	−1.748	0.086
PSS PPI	21.03 ± 17.66	41.07 ± 19.56	−4.196	0.000

*DUP* duration of untreatment psychosis, *PANSS* Positive and Negative Syndrome Scale, *PSC PPI* perceived spatial co-location PPI, *PSS PPI* perceived spatial separation

### Correlations among CT, sensory gating and symptoms

There were no significant correlation among CT, PPI and symptoms after the multiple comparison correction (Table 2). Furthermore, we did not observe significant associations between CT and PPI after the multiple comparison correction (Table 3).

**Table 2:** The relationship among childhood trauma, PPI, and symptoms of schizophrenia

	Positive	Negative	General Psychopathology	PANSS Total
Childhood trauma				
Emotional abuse	0.002	0.060	−0.126	−0.038
Physical abuse	0.317	0.143	0.186	0.312
Sexual abuse	−0.038	0.198	0.124	0.149
Emotional neglect	0.456 (0.005)	0.075	−0.259	0.131
Physical neglect	0.284	0.399 (0.018)	0.071	0.455 (0.008)
Total score	0.366 (0.036)	0.185	−0.132	0.220
PPI index				
Startle	−0.113	−0.340	− 0.011	−0.210
PSC PPI120	−0.373	−0.185	− 0.131	−0.240
PSS PPI120	−0.466 (0.004)	−0.231	− 0.302	−0.403
PSC PPI60	−0.216	−0.191	− 0.136	−0.223
PSS PPI60	−0.290	−0.322	− 0.207	−0.369

*PSC PPI* perceived spatial co-location PPI, *PSS PPI* perceived spatial separation, *PPI* prepulse inhibition

**Table 3:** The relationship between the specific of CT and PPI in first-episode schizophrenia

	Startle	PSC PPI120	PSS PPI120	PSC PPI60	PSS PPI60
Emotional abuse	0.194	−0.268	− 0.139	−0.591 (0.006)	−0.236
Physical abuse	−0.119	0.024	−0.507 (0.019)	−0.146	− 0.206
Sexual abuse	−0.036	0.021	−0.289	− 0.239	−0.354
Emotional neglect	−0.287	−0.064	− 0.203	−0.370	− 0.193
Physical neglect	0.016	−0.053	−0.258	− 0.338	−0.371
Total score	−0.112	−0.095	− 0.378	−0.495 (0.026)	− 0.394

*PSC PPI* perceived spatial co-location PPI, *PSS PPI* perceived spatial separation PPI, *CT* childhood trauma, *PPI* prepulse inhibition

### Regression analyses for CT subtypes, demographic features and PSS PPI

The multiple linear regression analyses showed that total score of CTQ-SF was negatively correlated with PSS PPI (ISI = 120 ms) (*p* = 0.018) (Table 4). We did not identify any type of trauma significantly correlated with either PSC PPI or PSS PPI (ISI = 60 ms) (data not shown).

**Table 4:** Multiple linear regression analyzing the impact of demographics, childhood trauma on PSS PPI (ISI = 120 ms)

PPI subscale	Odds ratio	95% CI	*p*
PSS PPI			
Constant	71.108	3.660~ 70.814	0.011
Childhood trauma	−1.542	−2.775~ − 0.309	0.018
DUP	0.431	0.021~ 0.840	0.041

R2 = 0.531, Adjusted R2 = 0.452, F = 6.784, *P* = 0.011

*DUP* the duration of untreatment psychosis, Dependent variables: continuous and normally distributed: Independent variables: Categorical variable: sex: 1. male 2. female; Smoke history: 1.No 2.Yes; the rest of variable are continuous: age, dup, education years; *PSS PPI* perceived spatial separation prepulse inhibition;

## Discussion

We examined the effect of CT on PPI in patients with first-episode schizophrenia (course of illness was less than 60 months). Overall, the patients had significantly higher score on SA and had more PPI deficit than healthy controls. More importantly, we found that CT was negatively correlated with PPI in the regression analysis.

CT has been demonstrated to have an impact on adult mental health, and exposure to early trauma has been linked to many psychopathologies, including schizophrenia [4]. In the current study, we found that patients with first-episode schizophrenia experienced higher level of SA. A number of studies in a range of samples attest to a link between childhood SA and psychosis, and SA before the age of 16 was strongly associated with schizophrenia, particularly if it involved non-consensual sexual intercourse [25]. A possible mechanism may be demonstrated via a neurodevelopmental model. Effects of SA on neurodevelopmental growth of the individual may give rise to neurocognitive deficits, further leading to the occurrence of schizophrenia [26]; We also found the patients had more severe PPI deficit than healthy controls, which is consistent with findings of other studies [7, 9, 27].

In the current study, we observed a negative correlation between CTQ-SF total and PSS PPI in patients. Evidence from animal studies may shed some light on the mechanism of exposure to traumatizing experiences on startle response and psychotic symptoms. One study, based on an experimental animal model, found that early life adversity during puberty was associated with increased risk for mental illness through sensory gating deficits [16]. Other study found that social isolation tend to have effects on PPI in rats [28]. Moreover, CT was associated with increased startle response in human [29]. Our findings showed that CT was indeed associated with sensory gating in first-episode schizophrenia, which attributed to the impact of early life adversity on neurodevelopment [26].

CT in humans shares some similarities with the socially isolated rat. At the behavioral level, social isolation induces hyperlocomotion, and dysfunctions in conditioned learning, reversal learning, and memory. [30]. At the endophenotype level, social isolation induces abnormalities in startle reflex and PPI. Moreover, social isolation causes changes of neurotransmitters, such as the increase of dopamine in the nucleus accumbens, the amygdala and other brain regions in the limbic system, the decrease of dopamine in medial prefrontal cortex, the decrease of 5-HT in the nucleus accumbens and the hippocampus, and changes of glutamine in the prefrontal cortex [30]. The proposed traumagenic neurodevelopmental model of schizophrenia shares similarities between the impact of CT on the brain development and the neurological abnormalities found in schizophrenia [26]. And, PPI is an important index which can reflect sensory gating function and the neurodevelopment [7]. So, we speculated that CT in human may be the risk factors to the occurrence of schizophrenia through its effects on sensory gating, and CT could cause a gene-environment interaction that result in the expression of schizophrenic symptoms.

This study has a few limitations: (1) The current study was a case-control study, a follow up study may be needed in the future to test the effect of CT on the development of sensory gating. (2). Psychotropic drugs with sedative effect have effects on PPI. In this study, 32 patients have taken drugs, which may have some influence on the results. (3). CT is deeply associated with both child factor and parental factor (for example parents’ neurodevelopmental disorder or psychiatric disease). Furthermore, it is suggested that subjects with sub-clinical autistic traits might have impaired sensory gating from birth. Unfortunately, we have not collected the information about developmental course, thus the possibilities that schizophrenic patients might have comorbid developmental disorders (especially ASD), which lead to impaired sensory gating observed in patients group.

## Conclusion

The patients with first-episode schizophrenia experienced a higher level of SA and deficit of sensory gating. Furthermore CT have effects on sensory gating, which may be contribute to the onset of schizophrenia.

## Abbreviations

ASR: Acoustic startle reflex
CT: Childhood trauma
CTQ-SF: The Childhood Trauma Questionnaire-Short Form
CWAIS: The Wechsler Adult Intelligence Scale
EA: Emotional abuse
EN: Emotional neglect
ISI: Inter-stimulus interval
PA: Physical abuse
PANSS: Positive and Negative Syndrome Scale
PN: Physical neglect
PPI: The prepulse inhibition
PSC PPI: The perceived spatial co-location PPI
PSS PPI: Perceived spatial separation-induced repulse inhibition (PSS PPI)
SA: Sexual abuse
SCID: The Structured Clinical Interview for DSM-IV
